# Differences in the production of hyperglycosylated IFN alpha in CHO and HEK 293 cells

**DOI:** 10.1186/1753-6561-7-S6-P33

**Published:** 2013-12-04

**Authors:** Agustina Gugliotta, Marcos Oggero-Eberhardt, Marina Etcheverrigaray, Ricardo Kratje, Natalia Ceaglio

**Affiliations:** 1 Cell Culture Laboratory, School of Biochemistry and Biological Sciences, Universidad Nacional del Litoral. Ciudad Universitaria - C.C. 242 - (S3000ZAA) Santa Fe, Provincia de Santa Fe, Argentina

## Background

IFN alpha is an important cytokine of the immune system. It has the ability to interfere with virus replication exerting antiviral activity. Moreover, it displays antiproliferative activity and can profoundly modulate the immune response. IFN4N (or hyperglycosylated IFN alpha) is an IFN-alpha2b mutein developed in our laboratory using glycoengineering strategies. This molecule contains 4 potential *N-*glycosylation sites together with the natural *O*-glycosylation site in Thr106 [1]. The resulting *N- *and *O*-glycosylated protein shows higher apparent molecular mass and longer plasmatic half-life compared to the non-glycosylated IFN-alpha produced in bacterial systems and used for clinical applications. As a consequence, the correct glycosylation of our modified cytokine is very important for its *in vivo *activity. For this reason, it is of great relevance the evaluation of different mammalian host cells for its production. While hamster-derived CHO cells are widely used for large scale production of recombinant therapeutic glycoproteins, human HEK cells are a promising system because they are easy to grow and transfect [2]. In this work, we performed a comparison between both production systems in terms of cell growth, culture parameters and specific productivity of hyperglycosylated IFN alpha.

## Results

**Figure 1:**
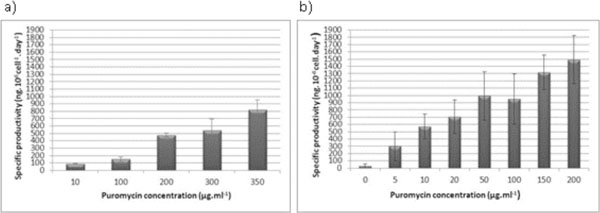
**Comparison between the specific productivity of the CHO-IFN4N (a)** and HEK-IFN4N **(b)** producing cell lines as a function of puromycin concentration.

Lentiviral vectors containing the sequence of IFN4N were assembled and employed for the transduction of CHO-K1 and HEK 293T cells. The recombinant cell lines were subjected to a process of selective pressure using increasing concentrations of puromycin. The CHO-IFN4N and HEK-IFN4N producing cell lines resistant to the highest concentration of puromycin showed the highest productivity of IFN4N. In particular, the CHO-IFN4N cell line was resistant to 350 μg/ml of puromycin and it showed a specific productivity of 817 ± 134 ng.10^6^cell^-1^.day^-1^, which represents an 8-fold increment compared to the parental line. The HEK-IFN4N cell line was resistant to 200 ug/ml of puromycin and showed a 15-fold increment in the specific productivity compared to the parental line, reaching a value of 1,490 ± 332 ng.10^6^cell^-1^.day^-1^. In both cases, complete culture death was achieved at higher puromycin concentrations. The specific productivity of IFN4N of HEK 293T cell line duplicated the value obtained for the CHO-K1 cell line, and it was achieved at a lower concentration of puromycin, making the selection process shorter (Figure 1).

Both cell lines were cloned using the limiting dilution method, and after 15 days of culture more than 100 clones were screened. To achieve the characterization and study both cell lines as recombinant protein expression hosts, the 6 best producer clones were isolated and amplified. The adherent clones were grown for 7 days in order to construct their growth curves. Cell density and viability were determined every 24 h by trypan-blue exclusion method and the culture supernatant was collected to determine IFN4N and metabolites concentration. The IFN4N production was assessed employing a sandwich ELISA assay developed in our laboratory. Glucose consumption and lactate production were evaluated using specific Reflectoquant^® ^test strips (Merck Millipore) in a RQflex^® ^Reflectometer (Merck Millipore). Levels of amonium in the culture supernatant were determined by the Berthelot reaction.

As shown in Table 1, the average specific growth rates of CHO and HEK clones were similar. However, CHO clones reached higher maximum cell densities (between 7.10^5^-1.5.10^6 ^cell.ml^-1^) than HEK clones (between 6.10^5^-9.10^5 ^cell.ml^-1^), probably because of space limitation and higher glucose consumption, since average q_gluc _of HEK clones was higher (see Table 1). No differences were observed between lactate and ammonium production of both groups of clones. In contrast, specific production rate of IFN4N was higher for the clones derived from the human cell line. Moreover, higher average IFN4N cumulative production for HEK clones was achieved after 7 days of culture (3,494 versus 5,961 ng.ml^-1^).

**Table 1:** Determination of the specific cell growth rate, specific production rate of lactate, ammonium and IFN4N, and specific consumption rate of glucose of CHO-K1 (a) and HEK 293T (b) clones.

a)															
Clones	μ(h^-1^)			q_IFN_ (ng.10^-6^cell.h^-1^)			q_gluc_ (μg.10^-6^cell.h^-1^)			q_lac_ (μg.10^-6^cell.h^-1^)			q_amon_ (nmol.10^-6^cell.h^-1^)		
**P4D3**	0,0182	±	0,002	79	±	8	36	±	5	40	±	5	0,027	±	0,007
**P1E9**	0,0196	±	0,002	35	±	4	24	±	4	45	±	5	0,027	±	0,003
**P2A9**	0,0249	±	0,001	41	±	4	30	±	2	31	±	1	0,014	±	0,004
**P1B6**	0,0240	±	0,002	19	±	3	26	±	5	49	±	5	0,013	±	0,005
**P1B7**	0,0191	±	0,002	41	±	3	32	±	4	35	±	2	0,017	±	0,006
**P1B8**	0,0277	±	0,002	42	±	3	21	±	3	34	±	2	0,015	±	0,004

**b)**															
**Clones**	**μ(h^-1^)**			**q_IFN_** **(ng.10^-6^cell.h^-1^)**			**q_gluc_** **(μg.10^-6^cell.h^-1^)**			**q_lac_** **(μg.10^-6^cell.h^-1^)**			**q_amon_** **(nmol.10^-6^cell.h^-1^)**		

**P2A5**	0,020	±	0,001	129	±	10	56	±	6	37	±	4	0,014	±	0,003
**P2C7**	0,015	±	0,002	122	±	13	62	±	11	38	±	3	0,009	±	0,003
**P2G11**	0,017	±	0,002	82	±	6	47	±	9	31	±	3	0,008	±	0,002
**P3B7**	0,016	±	0,002	99	±	8	55	±	8	31	±	3	0,008	±	0,003
**P3H8**	0,027	±	0,001	82	±	11	46	±	6	34	±	3	0,008	±	0,001
**P4B4**	0,017	±	0,002	63	±	5	61	±	15	32	±	3	0,009	±	0,001

## Conclusion

CHO and HEK cells were genetically modified to produce IFN4N by using lentiviruses as a tool for the IFN4N gene transfer. Since both cell lines expressed high levels of IFN4N, 6 clones were amplified for an intensive characterization. Culture and production properties of both groups of clones were very different. On the one hand, CHO clones were easy to maintain in culture for a long period of time, reaching higher cell densities than HEK clones. On the other hand, the best specific productivity of IFN4N was achieved employing HEK cells. The behavior of CHO and HEK cells at large scale production should be analyzed in order to select the proper system for the cytokine's production.

Wide differences have been observed between the glycosylation profile of the same recombinant therapeutic protein produced in CHO and HEK systems [2]. Considering that glycosylation affects protein bioactivity, stability, pharmacokinetics and immunogenicity, it would be very important to evaluate the characteristics of the IFN4N produced in both hosts to determine their efficacy as therapeutic agents.
